# An Expert Opinion on Hyaluronic Acid Fillers for Indian Facial Aesthetics: Insights From a Pre-meeting Questionnaire and Ad-Board Discussion

**DOI:** 10.7759/cureus.94079

**Published:** 2025-10-07

**Authors:** Rajat Kandhari, Chiranjiv Chhabra, Jagadish Sakhiya, Maya Vedamurthy, Ishad Aggarwal, Viral Desai, Pankaj Chaturvedi, Hema Pant, Rajetha Damisetty, Monisha Kapoor, Mikki Singh, Shweta Sawalka, Dyotona Sen, Biswajit Aich, Sameer Jadhwar

**Affiliations:** 1 Aesthetic Dermatology, Dr Kandhari’s Skin and Dental Clinic, New Delhi, IND; 2 Aesthetic Dermatology, Alive Welness, New Delhi, IND; 3 Aesthetic Dermatology, Sakhiya Skin Clinic, Surat, IND; 4 Aesthetic Dermatology, Rathnasabapathy Sitalakshmi Vedamurthy (RSV) Laser and Skin Centre, Chennai, IND; 5 Aesthetic Dermatology, Wizderm Speciality Skin and Hair Clinic, Kolkata, IND; 6 Aesthetic Dermatology, Cosmetic Plastic Laser Super Specialty (CPLSS) Sarla Hospital and Desai Hospitals Pvt Ltd., Mumbai, IND; 7 Aesthetic Plastic Surgery, Medlinks Clinic, New Delhi, IND; 8 Aesthetic Plastic Surgery, SCULPT Aesthetic and Cosmetic Clinic, New Delhi, IND; 9 Aesthetic Dermatology, Dr. Rajetha's Mohana Skin and Hair Clinic, Hyderabad, IND; 10 Aesthetic Dermatology, Dr. Monisha Kapoor Aesthetic, New Delhi, IND; 11 Aesthetic Dermatology, BodyCraft Salon, Bengaluru, IND; 12 Aesthetic Dermatology, Allure Medspa, Mumbai, IND; 13 Medical Affairs, Galderma India Pvt Ltd, Mumbai, IND

**Keywords:** beauty standards, facial aesthetics, hyaluronic acid fillers, indian aesthetics, indian facial characteristics, infraorbital hollows, nasolabial folds, restylane, skin laxity

## Abstract

This expert opinion explored aesthetic concerns among Indian participants, consolidating insights on physicians’ clinical needs assessments with patients’ self-identified concerns. Data was gathered through a pre-meeting survey distributed to 12 respondents. The findings revealed that physicians and patients consistently prioritize areas such as the infraorbital hollow in younger participants, while concerns shift toward volume loss and skin laxity in older age groups. Physicians often emphasize structural changes, such as malar bone resorption and jawline contouring. In contrast, patients frequently express concerns about visible signs of aging, including nasolabial folds, marionette lines, and neck skin laxity. The Assessment, Anatomy, Range, and Treatment (AART™) and Holistic Individualized Treatments (HITs™) enable practitioners to conduct thorough facial assessments and create tailored treatment plans. Considering both anatomical changes and patient expectations, an individualized approach to facial aesthetic treatments is necessary. There is a need for Indian-specific aesthetic guidelines owing to limited published literature that respects cultural facial characteristics and evolving beauty standards across age groups.

## Introduction and background

Maintenance of a pleasant, youthful, and aesthetic physical appearance is gaining attention as individuals age. This focus reflects a broader societal emphasis on the perception of beauty and its role in aging [1]. The “divine proportion,” also known as the golden ratio or phi (φ), is geometrically defined as 1:1.618 and is often considered the ideal ratio for aesthetic appeal [2]. While the classical “golden ratio” (φ) has historically informed artistic ideals of symmetry and balance, contemporary facial‐aesthetic practice emphasizes preserving each patient’s unique asymmetries. Rather than a strict target, φ can serve as a conceptual guide where clinicians instead tailor treatment to individual anatomy and aesthetic goals [3]. A single universal golden proportion is not indicative of underlying natural facial esthetics; facial harmony arises from variable, population-specific proportions rather than a single codified ratio [4]. While some aspects of facial beauty are universal, aesthetic preferences vary by ethnicity and culture, reflecting differences in facial bone structure, morphology, and skin tones [5]. In recent years, the popularity of minimally invasive aesthetic procedures has surged due to patients desiring options that deliver natural-looking results with minimal recovery time. For instance, the number of injectable botulinum toxin procedures has risen by 621% over the past two decades, highlighting a shift from surgical to non-surgical cosmetic interventions [1].

Soft tissue hyaluronic acid (HA) fillers have gained widespread acceptance among these non-surgical options. The United States Food and Drug Administration has approved fillers for lip, cheek, and chin augmentation applications [6-10]. According to the International Society of Aesthetic Plastic Surgery (ISAPS) 2023 report, the two leading non‐surgical procedures performed by plastic surgeons worldwide were botulinum toxin injections (8,877,991 procedures, 46.3% of all non‐surgical treatments; a 41.6% increase since 2019) and HA fillers (5,564,866 procedures, 29.0% of all non‐surgical treatments; a 28.9% increase since 2019) [11]. In India, soft tissue fillers are regulated as Class C medical devices under the Indian Medical Devices Rule (2017). They are primarily indicated for mid-to-deep dermal injections to correct wrinkles [12]. HA fillers, in particular, are favored due to their biocompatibility, which minimizes immunogenic reactions [13]. Fillers primarily serve two purposes: correcting dermal depressions to smooth wrinkles and adding volume to the facial skin and soft tissues. Patients generally report high satisfaction with the long-lasting effects of filler injections, which allow for subtle and sustained enhancement without frequent touch-ups, maintaining improved appearance over time without a complete reversion to pre-injection states [14]. They provide a durable yet non-permanent option for addressing age-related facial volume loss. By restoring volume, HA fillers help create a lifted, balanced, more harmonious appearance, allowing the face to be rejuvenated and delivering subtle and natural outcomes in the right hands [15].

The demand for facial treatments is rising and is influenced by the unique anatomical, cultural, and aesthetic preferences of the Indian population. Additionally, this is due to more disposable per capita income and pressures at work and from social media to “look a certain way.” This necessitates a region-specific approach to the use of HA filler. Given this background, an ad-board meeting among Indian dermatologists and plastic surgeons was held, in which the role of HA fillers in the Indian beauty context and clinical practices was discussed. The primary objective of this expert opinion was to consolidate insights from leading practitioners on best practices in HA filler selection and application tailored to the Indian context.

## Review

Methodology

Expert Panel Selection and Composition

A multidisciplinary expert panel was convened, comprising 12 key opinion leaders (KOLs) in aesthetic medicine, including dermatologists and plastic surgeons across India. The KOLs were selected based on their clinical experience, geographic representation (covering major regions of India), and academic contributions to the field. The panel represented major metropolitan cities, including Delhi, Mumbai, Bangalore, Chennai, Hyderabad, Ahmedabad, and Kolkata, providing a comprehensive national perspective on facial aesthetics in the Indian population.

Pre-meeting Activities

Before the advisory board meeting, a structured questionnaire was distributed to all participating experts to gather insights and set the discussion framework. The questionnaire comprised 11 questions designed to gather insights on the topic and set the context for the advisory board meeting (Appendix 1). Areas such as clinical practice patterns, facial assessment methodologies, decision-making for treatment, and specific clinical scenarios, such as approaches to heavy lower faces, management of tissue descent patterns specific to Indian faces, treatment strategies for nasolabial fold correction, patient selection, and protocols for Restylane® Skinboosters^TM^ and acne scar management were assessed. The questionnaire is presented for reference (Appendix 1). The questionnaire utilized multiple formats, including open-ended questions for detailed responses, Likert scale ratings for assessing utility, binary response questions for specific clinical approaches, and case-based scenarios for treatment planning. The responses helped identify and set the context, structure the advisory board agenda, and develop focused discussion points for the panel advisory board sessions.

Advisory Board Meeting Structure

The meeting was conducted in August 2024, following a structured format consisting of formal presentations and two moderated panel discussions. Formal presentations included topics such as understanding Indian beauty standards, the Facial Assessment Scale (FAS™), the Assessment, Anatomy, Range, and Treatment (AART™), rheological properties and clinical implications of HA fillers, product-specific considerations and positioning, and complementary aesthetic treatments with Restylane® Skinboosters™. In formal presentations, the initial presentation of evidence included reviews of current literature, clinical data presentations, and the sharing of expert experiences by a KOL. In the panel discussion segments, discussions on the questions were moderated by the moderator. Each expert's opinion was documented and then presented as an expert view.

Data Collection

Data collected included pre-meeting questionnaire responses, detailed minutes of all presentations, transcripts of panel discussions, post-discussion summaries, clinical photographs, and treatment approaches shared by experts. The entire process was documented through audio/video recordings of the sessions.

Results

The pre-meeting questionnaire responses from 12 experts on current facial aesthetic practices and preferences are detailed in Table 1.

**Table 1:** Responses obtained for the pre-meeting questionnaire 3D: Three-dimensional; AART™: Assessment, Anatomy, Range, and Treatment; CO_2_: Carbon dioxide; EBD: energy-based device; FAS™: Facial aesthetic scale; G': elasticity modulus (indicating the firmness or lifting capacity of fillers); HA: hyaluronic acid; HIFU: high-intensity focused ultrasound; MNRF: microneedling radio frequency; RF: radiofrequency.

Q No.	Area of discussion	Responses obtained
1	Primary patient concerns	Both male and female patients commonly seek consultations and treatment for anti-aging concerns, such as fine lines, sagging, and pigmentation. In addition, they often seek treatments for acne and acne scarring, skin-quality enhancement, and refinement of facial features, particularly for the lips, jawline, cheeks, and chin.
2	Facial assessment tools	Clinical examination is followed by the AART™ methodology, supported by dermascopes, FAS™, advanced 3D imaging, and Golden Ratio Caliper Arthur Swift measurements for precise assessment.
3	Utility of FAS™	On a scale of 1-5, 50% of the expert panel rated the FAS™ as useful, with 25% finding it useful in practice.
4	Age patterns	Patients typically approach the specialist for aesthetic treatments after attaining 30 years of age, with younger individuals often seeking feature enhancement and older patients focusing on volume restoration and sagging correction.
5	Differences in treating younger vs. older faces	Younger patients primarily approach the specialists for feature enhancements (e.g., lips, nose, chin, cheeks, and tear trough corrections) and soft tissue augmentation. Older patients typically seek improvement in sagging, skin quality, volume restoration, correction of folds and wrinkles, along with rejuvenation of the periorbital area and the neck. Treatment areas often include the periorbital area, skin quality, neck, lip inversion, and wrinkles. Older patients may require combination treatments or surgical interventions due to greater skin laxity and volume deficits.
6	Preferences for HA fillers	Decisions are influenced by gel properties (firmness, flexibility, HA concentration, degree of modification, and enzyme degradation responsiveness) and product clinical performance (effectiveness, safety, and natural-looking results).
7	Approach to Indian facial structures and tissue descent	As per the individual’s need, HA filler use is prioritized. Experts also initiate treatments with EBDs and threads, followed by fillers for volume-deficient areas. Healthcare professionals recommended contouring the outer face first (temples, zygomatic region, preauricular area, pre- and post-jowl area, gonial angle, and chin), lifting the face with fillers, combined with skin-tightening methods such as radiofrequency, ultrasound-based devices (particularly for the mid face), and threads if needed for a comprehensive correction. Suggested techniques include debulking with liposuction, lipolysis, or HIFU, combined with high G' fillers for targeted lifting.
8	Approaches for heavy lower faces	Foremost, underlying issues (e.g., masseter hypertrophy, parotid prominence, general heaviness, or skin laxity) must be assessed before formulating a tailored treatment plan. Targeted interventions include fillers for reshaping the chin and jawline, marionette lines, and pre-jowl areas. Botulinum toxin should be applied to the platysma, mentalis, depressor anguli oris, and hypertrophic masseter muscles along with parotid hypertrophy. In addition, lipolysis for submental fat reduction may be considered.
9	Nasolabial fold correction	Opinions were divided on whether nasolabial fold correction should be a fundamental approach. Half of the experts favored nasolabial fold correction as a base for lifting the midface, while others preferred a global assessment to treat root causes for more natural results.
10	Ideal candidates for Restylane^®^ Skinboosters™	Restylane^®^ Skinboosters™ is suitable for patients looking for subtle changes, often combined with treatments such as peels, lasers, and RF tightening to enhance hydration, glow, and skin quality.
11	Restylane^® ^Skinboosters™ for acne scars	Combining Restylane^®^ Skinboosters™ with trifarotene (a topical retinoid) has shown benefits for atrophic acne scars. However, experts also recommend pairing with MNRF and CO₂ for optimal, event-ready results with repeated sessions for sustained improvements.

Discussion

Understanding Indian Facial Morphology and Beauty Standards

The perception of facial beauty is universally similar; however, ethnicity, cultural differences, and trends make the idea of facial attractiveness vary in different regions. Indian facial morphology typically exhibits a more convex profile with the prognathic maxilla, mandible, and chin, along with more protrusive upper and lower incisors in the horizontal dimension. In the vertical dimension, they exhibit a decreased middle third facial height as well as a decreased ramal and corpus length of the mandible when compared with the Caucasians [16]. Sexual dimorphism further shapes beauty ideals, where men’s attractiveness in India often rests on a strong upper‑face structure and a defined jawline. In contrast, women’s attractiveness emphasizes mid‑face fullness, soft cheek contours, and graceful jaw angles [16]. Indian beauty ideals often favor a well-defined nasal bridge with projection, fuller lips and cheeks, and fair, or at least even skin tone [3,17].

India’s diversity in culture, climate, and regional characteristics contributes to varied facial shapes and skin tones, which influence aesthetic needs. Regional differences in facial structure and aging patterns, such as a more pronounced descent of facial tissues and an earlier onset of age-related changes, such as losing suborbicularis oculi fat from their early 20s as compared to Caucasians, necessitate tailored approaches to aesthetic interventions [18]. The aesthetic appeal of an oval face shape is universally acknowledged and helps guide aesthetic treatments, defining areas to enhance in younger patients and restore aging faces [19,20]. Historically associated with beauty, the golden ratio does not consistently apply to natural facial ratios across populations. Research has shown deviations from this ratio in facially attractive individuals, particularly in Indian facial proportions [4].

The optimal facial proportions adhere to the “Rule of Thirds and Fifths,” which divides the face into five vertical sections to maintain balanced facial width (reference to the neoclassical canon of facial proportions). Additionally, the width of each eye is proportionate to the intercanthal distance. In India, ideal facial aesthetics emphasize an oval face shape with prominent cheekbones, a defined jawline, and a balanced Ogee curve. However, the Indian facial structure presents unique challenges due to its smaller facial bone frameworks; differences in skeletal shape, size, and soft tissue disposition; wider, fuller faces than Caucasians; and the natural descent of facial tissues over time [5]. Furthermore, aging in Indian individuals often manifests as the development of tear trough, midface atrophy, deepening of the nasolabial fold, hollow temples, and lip thinning. As aging progresses, Indian faces tend to become fuller and the tissues descend downwards and medially; a descent often more pronounced due to a higher volume of facial fat pads and smaller bone framework [5,21,22]. Indian facial anatomy, characterized by a smaller bony structure and relatively abundant superficial fat, demonstrates an early pattern of fat loss and descent in the lateral superficial compartments, particularly the temporal and preauricular (periauricular) pads. Hence, volumetric restoration should therefore prioritise temporal and preauricular augmentation (deep or superficial, depending on the plane of depletion) to restore facial harmony without producing anterior crowding [23]. Age-related aesthetic priorities vary; younger Indian women frequently report infraorbital hollows as a concern, while older groups are more concerned with nasolabial folds, marionette lines, and skin laxity. Physicians generally focus on malar volume loss and upper facial lines as age advances. Filling the preauricular region with an appropriately calibrated volume of filler reduces the visible prominence of jowls and marionette lines, thereby lessening the amount of filler required in the lines themselves and in the prejowl sulcus [5,21,22]. A soft tissue disposition is common in Indian individuals and can often be corrected with soft tissue fillers alone, although it can be challenging. Anthropometric studies indicate that Indian faces tend to have a smaller total face height, a proportionately larger forehead, and more prominent, widely spaced eyes (a rounded facial shape). The lower face is often wider and shorter with a smaller and retruded chin, commonly resulting in mentalis hyperactivity and hypertrophy, due to excess medial soft tissue. Although Indians have a structurally smaller and narrower midface, the presence of excess medial soft tissue means restoration of age-associated volume deficit is usually required in the lateral midface [5]. Masseter hypertrophy also contributes to a shorter lower one-third of the face compared with upper and middle one-thirds, affecting facial harmony in Indians [5,21]. Anthropometric data from Indian populations demonstrate clear sexual dimorphism in the upper and lower facial thirds, which has practical implications for HA-filler planning. Indian men generally exhibit larger vertical and horizontal facial dimensions in the upper and lower thirds, a relatively wider palpebral fissure, and a more laterally positioned brow apex (~1-2 mm lateral to the lateral canthus), whereas women commonly display relatively smaller upper-third dimensions and a facial attractiveness strongly influenced by lower-face (mandibular and chin) contours. Men also tend to have longer, wider noses with increased tip projection and a narrower nasofrontal angle; conversely, vertical upper-lip height and oral-commissure width are greater in men than in women. These sex-specific structural differences, particularly the prominence of the upper half in determining male attractiveness and the lower half (mandibular contour) for female attractiveness, should guide filler selection and placement [24].

Studies examining lips often explore concepts of attractiveness through aesthetic evaluations or guidelines aimed at achieving ideal augmentation results. A meta-analysis of the lower face and lips in healthy Caucasian populations has indicated that attractiveness is closely tied to the harmony of proportions in this area. Fuller, more prominent lips are typically perceived as more appealing for both men and women. Notably, the morphometric features of the lower face undergo significant changes with aging, altering these attractive qualities over time [25]. Studies show no universal lip ratio; the often-cited 1:2 upper-to-lower lip height in Caucasians varies ethnically. Among young Caucasians, an upper-to-lower lip height ratio of approximately 1:1.6 is considered attractive. In contrast, Indian populations typically exhibit thinner upper vermillion, shorter upper-lip length, and a different lip-to-face width proportion than Caucasians [26,27]. HA fillers are commonly used to address these age-related changes, particularly in the lips and chin; however, overuse may lead to deformities [1]. Hyperpigmentation is a primary concern among the Indian population, generally triggered or worsened by sun exposure. Indians have fewer static lines due to good skin quality and are prone to periorbital hyperpigmentation, or dark circles, which affects approximately 50% of Indian women, with moderate-to-severe cases increasing with age [28]. Periorbital hyperpigmentation treatment should focus on identifying and addressing the primary cause and contributing factors. HA gel fillers are commonly used to reshape the periorbital area to enhance tear trough contour, achieving high patient satisfaction [29]. Table 2 summarises the expert opinions gathered during the meeting on Indian beauty standards and aesthetic concerns.

**Table 2:** Expert opinion on Indian beauty standards and aesthetic concerns

Indian faces exhibit unique and ethnic anatomical characteristics, including early onset of tear trough formation, periorbital hyperpigmentation, and generally heavier facial structures.
Psychological assessments of facial attributes are typically formed within 100 milliseconds of viewing a face. These assessments highly influence key aspects of beauty, such as symmetry, proportion, averageness, and youthfulness.
The ideal facial proportions follow the “Rule of Thirds and Fifths,” where facial width is balanced across five vertical sections, and each eye’s width matches the intercanthal distance. An oval face shape with smooth, continuous curves from the temple to the chin is most desirable in women.
Age-related aesthetic issues frequently observed include fat loss around the eyes, sagging, and a downward shift of facial tissues.
Indian patients commonly seek aesthetic enhancements for periorbital hyperpigmentation, chin projection, lip augmentation, and malar volume loss. Indian facial features often include a larger forehead, proportionally shorter lower face, and a unique morphotype characterized by a wider zygomatic arch, deep-set eyes, and early tear trough formation.
Facial aging in Indian individuals manifests as hypertrophic fat in the nasolabial, preauricular, and jaw areas, producing prominent nasolabial folds.
Men typically share similar structural traits as women but show less symmetry in bizygomatic and bigonial widths. Additionally, Indian faces tend to have smaller mid-faces, longer foreheads, and greater soft tissue accumulation, contributing to central facial heaviness and a tendency toward hyperpigmentation due to higher melanin levels.
Indian women’s most common aesthetic concerns include periorbital hyperpigmentation and tear trough deformities (ages 20–40 years), chin projection inadequacy and lip volume (ages 20–30 years), and malar volume loss (over 40 years), and a need for volume restoration.

The Indian population frequently expresses reservations regarding filler treatments, specifically HA fillers and volumizers, mainly due to concerns about repeated treatments, pain, and the potential for unnatural outcomes. A systematic review of 13 studies studying the impact of HA injectables on facial skin quality found that all HA formulations significantly enhanced skin hydration, firmness, brightness, texture, radiance, and elasticity while reducing signs of skin fatigue. The results showed that HA monotherapy produced more noticeable effects than treatments with other combinations such as HA and multivitamin solution, HA (non-cross-linked) and mannitol, HA (non-cross-linked high-viscosity) and multivitamin solution, recombinant epidermal growth factor with a filler grade HA, and a cocktail (multiple injections) of HA, vitamins, amino acids, minerals, coenzymes, and antioxidants. Additionally, HA treatments were safe, and high patient satisfaction was reported [30].

Facial Assessment Approach

Facial assessment has a strong psychological component, as personal attributes are often appraised instantaneously. A common approach to assessing facial symmetry involves dividing the face into thirds and fifths. Studies indicate that attractive female faces often display proportionality across these divisions, with balanced facial thirds and uniform lip features, significantly influencing perceptions of beauty [31]. Masculine faces are those with broader jaws, thicker brow ridges, and longer lower face halves. Men tend to rate masculinized faces as more dominant, emphasizing the role of facial sexual dimorphism [32,33]. Skin quality assessment is also relevant across all ethnicities, ages, and genders. It assesses skin tone uniformity, surface homogeneity, firmness, and glow [34]. Recently, 10 global experts collaborated to develop the Skin Quality Assessment Scale (SQS), which encompasses texture (pores, lines, and scars), discoloration (redness, pigmentation, and dullness), firmness (laxity), and hydro-lipid balance (oiliness and dryness). The SQS, validated by a large group of clinicians, scored high for comprehensiveness and practicality in clinics, aiding practitioners in setting treatment priorities and tracking skin improvements [35].

Two methodological tools, AART™ and Holistic Individualized Treatments (HITs™), enable practitioners to conduct thorough facial assessments and create tailored treatment plans. AART™ focuses on systematic facial evaluation, anatomical understanding, product selection, and holistic treatment planning, while HITs™ targets specific facial areas, allowing for the precise application of aesthetic products. Over 85% of surveyed clinicians found that the AART™-HITs™ methodology is adequate for clinical needs, with high satisfaction in temporal sequencing, anatomical accuracy, and product range, deeming the FAS™ diagnostic tool effective in personalizing treatment for diverse ages and ethnicities [36]. In anecdotal evidence, it has been noted that the Global Ranking Scale (GRS) serves as a guide to enhance consultation and patient satisfaction in facial rejuvenation treatments. Over 500 clinics globally use the GRS within Galderma’s Harmony Program, where clinical experiences show that GRS offers various advantages beyond patient assessment alone. Specifically, the scale provides flexibility in patient assessment and management, enhances precision in identifying treatment needs, fosters open dialogue between physicians and patients, strengthens rapport, builds trust, and encourages patients to self-reflect and communicate their goals and preferences. The GRS also helps patients understand the treatment plan, which can lower anxiety and improve satisfaction [37]. Table 3 summarizes the expert opinions gathered during the meeting on the Galderma facial assessment scale.

**Table 3:** Expert opinion on Galderma facial assessment scale FAS™: Facial assessment scale.

FAS™ supports a comprehensive approach to facial aesthetics, meeting the growing need for full-face evaluation even when patients initially present with isolated concerns. FAS™ provides a systematic and standardized method that not only aids in assessment but also actively engages patients in their aesthetic treatment journey.

Understanding the Clinical Implications of Rheological Properties and the Scientific Basis of HA Filler Selection

HA is a carbohydrate, a sugar molecule, that can bind large amounts of water and is crucial in maintaining skin hydration. With age or due to external factors, the skin's content of both HA and collagen decreases, making the skin thinner, sagging, and less hydrated [38]. HA fillers are often injected into deep layers beneath the superficial musculoaponeurotic system (SMAS), where pressure from ligaments and the SMAS affects filler shape retention. Softer fillers work well in this space but lack durability, while firmer fillers maintain form under pressure; they may raise risks such as foreign body sensation and impaired circulation. The filler used for a particular indication may be used in the appropriate layer for optimal outcomes as per its rheology. Thus, filler elasticity as well as rheology should match the injection layer for optimal outcomes [14]. The elastic modulus quantifies the strength or firmness of HA gels, denoted as G'. At the same time, flexibility is characterized by xStrain, representing the strain value at the G double prime (G'/G") crossover point during amplitude testing. This xStrain value indicates the gel’s recovery limit after deformation; beyond this threshold, the gel starts to behave more like a liquid and loses its ability to regain its original shape and form. Since G' and xStrain are distinct and unlinked properties, products with similar G' values can show different xStrain values and vice versa. Generally, gels with a higher G' are robust and less susceptible to deformation, whereas those with a higher xStrain are flexible [15].

The Restylane® (Galderma Laboratories, Fort Worth, TX, USA) line includes NASHA® and Optimal Balance Technology (Trademark) (OBT™) fillers, offering a range of firmness and flexibility to suit different facial regions. NASHA® provides distinct borders and minimal tissue integration, which is ideal for deeper injections in areas such as the cheek for lifting effects. At the same time, OBT™ fillers are more integrative and suited for dynamic facial areas or thin skin. In studies, NASHA® products (Restylane® and Restylane® LyftTM) demonstrated higher G' (701-799 Pa), indicating greater firmness for lift and projection. OBT™ products (Restylane® Kysse™, Restylane® Volyme™, and Restylane® Defyne™) showed lower G' (70-271 Pa) with high flexibility (up to 1442% xStrain), making them ideal for areas requiring flexibility, such as dynamic facial zones. Together, NASHA® and OBT™ fillers offer versatile options across strength and flexibility ranges. Restylane® Volyme™ is a softer, moderately pliable gel designed for gentle fullness and smooth contouring in areas like the cheeks and preauricular areas without heavy structural impact. Restylane® Defyne™ is a firmer gel with larger HA particles and higher G′, providing robust structural support for deeper folds, moderate-to-severe nasolabial lines, and defined chin and jawline contouring [15,39,40].

A study evaluating G' and xStrain for NASHA® and OBT™ fillers demonstrated distinct rheological profiles. Restylane® and Restylane® LyftTM, formulated using NASHA® technology, had high G' values (701 Pa, 416 Pa, and 799 Pa) but low xStrains (7%, 19%, and 17%). The NASHA® technology, used for Restylane® and Restylane® Lyft™, preserves the natural HA chain length. The NASHA® technology uses minimal modification, producing strong, firm gels optimal for lift and projection, particularly in areas requiring structural support, such as the nose and chin. In contrast, OBT™ fillers, including Restylane® Kysse™, Restylane® Defyne™, and Restylane® Volyme™, display lower G' values (70-271 Pa) but significantly higher xStrains (761%-1442%), making them softer, yet highly flexible. The OBT™ technology varies in G' through degrees of crosslinking, yielding gels that can deform with facial movements and return to shape afterward. This flexibility is particularly advantageous in dynamic areas, such as the nasolabial folds, marionette lines, and perioral regions, where frequent strain occurs due to facial expressions. The choice between NASHA® and OBT™ fillers depends on treatment goals. Stronger, firmer NASHA® gels are suitable for structural enhancements, whereas flexible OBT™ gels are ideal for dynamic areas where natural movement is essential. Softer gels with high xStrain can adapt to deformations during facial expressions, providing a more natural look and feel in areas subject to movement [15]. Table 4 summarizes the expert opinions gathered during the meeting on rheology and clinical implications for fillers.

**Table 4:** Expert opinion on rheology and clinical implications for fillers G': Elasticity modulus (indicating fillers' firmness or lifting capacity); OBT™: Optimal Balance Technology (Trademark).

NASHA^®^ products, such as Restylane^®^ Lyft™, with higher G', provide optimal structural support and are best suited for targeted lifting in the cheek and jawline.
OBT™ products such as Restylane^®^ Volyme™, Restylane^®^ Defyne™, and Restylane^®^ Kysse™, with lower G', offer flexibility and are ideal for dynamic, high-mobility areas, including lips and soft tissue; they are especially appropriate for patients with thin skin.
Patient satisfaction with Restylane^®^ products is high due to their natural-looking and durable results, particularly with Restylane^®^ Defyne™ for chin and perioral correction and Restylane^®^ Kysse™ for lip enhancement.
The Restylane^®^ range demonstrates higher G' and flexibility than competitor fillers, often requiring a lower volume to achieve the desired results.

Approaches to Specific Areas

Facial rejuvenation requires tailoring treatment strategies to the unique anatomical and cultural characteristics of the Indian face. Specific areas, such as the tear trough, nasolabial folds, malar region, chin, lips, and lower face, require individualized approaches. Factors such as skin thickness, dynamic movement, tissue descent, and the risk of complications (e.g., the Tyndall effect in thin periorbital skin) guide the choice of filler (with its corresponding rheological properties) and the injection technique. In Indian faces, the placement of fillers in the outer facial zones, rather than the inner ones, aligns with cultural aesthetics and fosters elegant aging [21]. Working on the outer zones of the face positively affects the diverse facial shapes seen in India: An outer-zone (or “circle”) injection can enhance facial definition in broader or more hypertrophic profiles by creating subtle skeletal refinement and angularity. Conversely, in individuals with more prominent bony frameworks, often encountered in some Northeast Indian populations, targeted softening of the lateral malar region can harmonize the overall contour. Because smaller, more delicate facial proportions are generally preferred, one should exercise caution when augmenting the outer facial zone with high-area fillers (e.g., around the lateral malar areas). Indians prefer small faces, so one should be cautious before adding hyaluronic acid fillers to the outer zone, especially around the lateral malar areas [21]. Selecting the appropriate filler, based on firmness (G'), flexibility (xStrain), and injection technique, is crucial for achieving natural-like, long-lasting results for each facial area. Table 5 below outlines approaches for these key facial areas.

**Table 5:** Concise treatment approaches for HA filler applications and product details (expert-recommended example products and approximate dosing) G': Storage modulus (measure of gel stiffness); DMCF: Deep medial cheek fat; HA: hyaluronic acid

Facial area	Patient characteristics and goals	Recommended approach and products
Tear trough	•Younger: Simple structural changes; no eye bags or midface deficit •Older: Volume loss, negative orbital vector, eye bags, and puffiness	•Younger: Direct correction with Restylane^®^ (1 mL total; 0.5 mL per side). To be repeated in 2-4 weeks for deep tear troughs to optimize outcomes •Older: Two-step approach: 1. Cheek support with Restylane^®^ Lyft™ (0.5-1 ml each side) 2. Tear trough correction with Restylane^®^ (0.5 mL per side via cannula) •Additional: Supraperiosteal injection with a high G’ filler; adjunctive midface volume lifting using Restylane^®^ Lyft™/Volyme™
Midface and nasolabial region	•Older: Sagging, ptotic, thick skin •Younger: Moderate-to-severe volume loss with thinner, pigmented skin	•Bony projection enhancement using Restylane^®^ Lyft™ (supraperiosteal injection) •Additional lifting for DMCF enhancement with Restylane^®^ Volyme™ •Marionette line correction with Restylane^®^ Defyne™ •For dynamic volume loss, Restylane^®^ Volyme^TM^ is recommended; skin quality improvement via NASHA^®^ Restylane^®^ Skinboosters™ Approximate dosing for the midface and nasolabial region: Midface: 2 mL per treatment session Nasolabial: 1–2 mL per session
Lower face, chin, and jawline	•Need for chin projection, symmetry, and jawline contouring	•Chin: Build the base with Restylane® Lyft™ on bone (needle preferred; cannula optional in elderly) plus refinement with Restylane^®^ Defyne^TM^ •Dosing: Female 2 mL; male up to 2 mL (the injection volume should be spaced across multiple treatment sessions) •Jawline: Contour with Restylane^®^ Lyft™ (up to 2 mL) and correct the gonial angle with 0.5 mL per side using an inverted cone technique.
Skin type considerations	•Thin skin: Requires a more flexible product •Thick skin: Needs robust structural support	• Thin: Use Restylane^®^ Defyne™ (OBT™) for flexibility. •Thick: Use Restylane^®^ Lyft™ for enhanced support.
Perioral area and lips	•Enhancement of perioral volume and lip definition	•Use Restylane^®^ Defyne™ for overall volume and structure. •Lips: Use Restylane^®^ Kysse™ (1 mL/session). •Injection technique: For thin lips, combine deep cannula (submucosal) with superficial needle injections; for normal-to-thick lips, direct needle injection is preferred. •Apply top-up sessions as needed.

*Complementary Aesthetic Treatment with Restylane® Skinboosters*™

The concept of Restylane® Skinboosters™ has transformed from solely enhancing skin volume with HA fillers to a broader focus on improving skin health and resilience. Now, products such as Restylane® Skinboosters™ Vital and similar HA fillers address aging signs and directly hydrate and support the dermis, improving elasticity and reducing fine lines. Restylane® Skinboosters™, non-cross-linked HA skin boosters, offer a more subtle volumizing effect and a shorter-lasting impact than cross-linked HA skin boosters. Traditionally, treatments such as microneedling or energy-based devices (EBDs) stimulate the dermis; however, Restylane® Skinboosters™ strengthens the dermal matrix without invasive techniques [41].

The application of Restylane® Skinboosters™ involves small amounts of minimal cross-linked HA being deposited into the dermis. They enhance the appearance of the skin by providing deep hydration and addressing signs of aging. HA Restylane® Skinboosters™ is available in two forms: non-cross-linked and low-cross-linked HA [41]. Restylane® Skinboosters™, such as Vital, deliver stabilized HA directly into the dermis to improve texture, reduce wrinkles, and stimulate collagen without affecting adjacent tissues [21,42,43]. The HA backbone is modified for enhanced longevity [38]. The hyaluronic acid chemical structure is modified, primarily by controlled cross-linking (type of cross-linker, degree of modification), HA concentration, and molecular weight to increase resistance to enzymatic degradation [44,45].

Energy-based devices (EBDs), including lasers, high-intensity focused ultrasound, and radiofrequency, are increasingly used for skin aging management by delivering thermal energy to deeper skin layers to promote tissue contraction and nucleogenesis, improving skin laxity and reducing wrinkles [46]. A study combining internal laser therapy with injectable Restylane® Skinboosters™ revealed enhanced skin tightening and elasticity, as the laser stimulates collagen and cellular rejuvenation. In contrast, Restylane® Skinboosters™ adds volume and strengthens the skin’s structure. The combination increases patient satisfaction compared to laser treatment alone, demonstrating a synergistic effect in skin rejuvenation [47]. Apart from facial aging, HA filler use is also recorded in other aesthetic corrections. In a placebo-controlled study, microinjections of stabilized HA gel in aging hands showed significant improvements in skin condition, hydration, elasticity, and roughness at three months compared to baseline (one month) and saline-treated hands, with effects sustained up to 12 months. The treatment was well tolerated, with only mild, anticipated adverse events. Patient scores and self-assessments were consistently higher for the HA gel than saline, indicating long-term efficacy in hand rejuvenation [48]. Similarly, a 15-month multicenter study in Chinese patients demonstrated that HA treatments for aging hands produced high satisfaction rates, sustained aesthetic improvements, and had well-tolerated effects [49].

The Corneometer® is recognized as a highly precise tool for spectroscopic measurement of skin hydration [50]. In a retrospective review of 20 subjects treated with small-particle hyaluronic acid (SP-HA) Restylane® Skinboosters™, hydration changes were evaluated using the Corneometer® CM 825 with the Multi-Probe Adapter. Initial measurements indicated “dry” hydration levels (30-40) for the face at Visit 1, which improved to “sufficiently hydrated” (>40) by Visits 2, 3, and 4, demonstrating the treatment with SP-HA Restylane® Skinboosters™ elevated hydration levels, changing the face from “dry” to “moisturized” and hands from “very dry” to “dry” [14,51]. Table 6 summarises the expert opinions gathered during the meeting on aesthetic treatment with Restylane® Skinboosters™.

**Table 6:** Expert opinion on aesthetic treatment with Restylane® Skinboosters™ EBD: Energy-based device.

Restylane^®^ Vital is recommended to improve skin tone and texture, and provide a lasting hydration effect, particularly in the cheeks, chin, neck, and hands: 3 treatments, 4 weeks apart, with maintenance every 6 months, is recommended.
Techniques such as micropuncture and linear injections are effective for Restylane^®^ Skinboosters™, providing a structured approach that combines needle and cannula applications.
Skin quality, assessed by firmness and elasticity, can be improved with EBDs and Restylane^®^ Skinboosters™ in older patients; younger patients may benefit from Restylane^®^ Skinboosters™ alone.
Restylane^®^ Skinboosters™enhances skin surface smoothness, tone, and glow and reduces fine lines, wrinkles, and scarring through various mechanisms.
Restylane^®^ Skinboosters™ Vital shows long-lasting results for both the face and aging hands, with no major safety issues.
Corneometer^®^ studies indicate that Restylane^®^ Skinboosters™ treatments transition facial skin from dry to moisturized and improve hand hydration from very dry to dry.
Noticeable facial improvement is typically achieved after the first of three Restylane^®^ Vital sessions, while hands require two sessions to reach visible hydration benefits.
Key benefits of Restylane^®^ Skinboosters™ include versatile indications, compatibility with other treatments, quick onset, long-lasting effects, easy administration, cost-effectiveness compared to fillers, minimal complications, no contraindications, and high patient satisfaction.

## Conclusions

The expert opinion provides insights into the growing beauty consciousness of the Indian population, which has unique aesthetic concerns, as its beauty standards are often distinct from Western paradigms. Influenced by cultural ideals and anatomical nuances, Indian aesthetics favor balanced facial proportions with specific regional traits, such as a heavier lower face, early tear trough formation, and distinctive patterns of hyperpigmentation, which together define a naturally youthful yet defined appearance. These concerns illustrate changes in priorities for aesthetic intervention with advancing age. The present expert opinion bridges the knowledge gap by providing valuable recommendations on the importance of HA filler usage in the Indian context. A critical observation from the panel discussion is the mismatch between healthcare professionals’ (HCPs) treatment goals and patients’ expectations. While HCPs typically focus on structural corrections and anatomical refinements, patients often prioritize visible improvements such as the reduction of fine lines, folds, and sagging skin. This mismatch calls for better communication and tailoring treatment strategies that align clinical interventions with patient desires in the best way possible. Furthermore, the present study recommends a comprehensive approach incorporating a full range of HA fillers. In addition to a range of structural HA fillers, with products available in various formulations offering different rheological properties (from high G' fillers that provide robust lift and support for areas like the midface and chin, to lower G' formulations ideal for dynamic regions), the present study encourages considering adjunctive treatments. Treatment strategies should also extend to Restylane® Skinboosters™, such as HA-based Restylane® Skinboosters™ Vital, which improves skin hydration and elasticity. The expert opinion acknowledges and addresses the specific aesthetic concerns in the Indian population and highlights the importance of patient-centric approaches to yield effective results and improved patient experience.

## Appendices

**Table 7:** Pre-meeting questionnaire distributed to the KOLs KOL: Key opinion leader; FAS™: Facial assessment scale.

Questions
In your clinical practice, what are the indications that patients (both female and male) visit you for facial aesthetic treatment?
Which tools do you use for a facial assessment?
On a scale of 1-5, how useful is the FAS™ in your clinical practice?
What is the common age group for men to approach you for facial aesthetic treatment vs. women?
What are the unique properties of hyaluronic acid fillers that impact your clinical decision for a particular indication?
Are there any significant differences between treating younger and older faces in your practice?
Data indicates Indian faces tend to get fuller, and tissues descend downwards and medially, making it complicated for aesthetic correction. How commonly is this seen in your clinical practice, and what is your approach to such faces?
What are your approaches for heavy lower faces?
Significantly, in the Indian population, the lower third of the face is much shorter than the upper and mid-third of the face. Is nasolabial fold correction acceptable as the most basic and effective approach in aesthetic correction?
Who are the ideal candidates for Skinboosters?
Data indicate that sequential treatment with trifarotene plus Restylane^®^ Vital has proven beneficial for atrophic acne scars. What are your views on the Skinboosters’ role in treating acne scars?

## References

[1] Li K, Meng F, Li YR (2022). Application of nonsurgical modalities in improving facial aging. Int J Dent.

[2] Ricketts RM (1982). The biologic significance of the divine proportion and Fibonacci series. Am J Orthod.

[3] Khan NA, Nagar A, Tandon P, Singh GK, Singh A (2016). Evaluation of facial divine proportion in North Indian Population. Contemp Clin Dent.

[4] Londono J, Ghasmi S, Lawand G, Mirzaei F, Akbari F, Dashti M (2024). Assessment of the golden proportion in natural facial esthetics: a systematic review. J Prosthet Dent.

[5] Kapoor KM, Chatrath V, Anand C (2017). Consensus recommendations for treatment strategies in Indians using botulinum toxin and hyaluronic acid fillers. Plast Reconstr Surg Glob Open.

[6] Coppini M, Caponio VC, Mauceri R, Pizzo G, Mauceri N, Lo Muzio L, Campisi G (2024). Aesthetic lip filler augmentation is not free of adverse reactions: lack of evidence-based practice from a systematic review. Front Oral Health.

[7] Galderma Laboratories, L.P L.P Restylane® Kysse: instructions for use. Galderma Laboratories, L.P. https://www.galderma.com/us/sites/default/files/2022-06/Restylane_Kysse_e-IFU_US.pdf.

[8] Galderma Laboratories, L.P L.P Restylane® Lyft with Lidocaine: instructions for use. https://www.galderma.com/us/sites/default/files/2019-01/Restylane_Lyft_with_Lidocaine_IFU.pdf.

[9] Galderma Laboratories, L.P L.P Restylane® Defyne: instructions for use. Fort Worth (TX): Galderma Laboratories Sweden.

[10] Galderma Laboratories, L.P L.P Restylane® Refyne: instructions for use. https://www.galderma.com/us/sites/default/files/2019-01/Restylane_Refyne_IFU.pdf.

[11] International Society of Aesthetic Plastic Surgery (ISAPS (2023). International Survey on Aesthetic/Cosmetic Procedures. International Society of Aesthetic Plastic Surgery (ISAPS). https://www.isaps.org/media/rxnfqibn/isaps-global-survey_2023.pdf.

[12] Lahiry S, Sinha R, Chatterjee S (2019). Medical device regulation in India: what dermatologists need to know. Indian J Dermatol Venereol Leprol.

[13] Czumbel LM, Farkasdi S, Gede N (2021). Hyaluronic acid Is an effective dermal filler for lip augmentation: a meta-analysis. Front Surg.

[14] Hong GW, Wan J, Chang K, Park Y, Yi KH (2025). Decomposition and changes in in vivo post-HA filler injection: a review. J Cosmet Dermatol.

[15] Ohrlund A, Winlof P, Bromee T, Prygova I (2024). Differentiation of NASHA and OBT hyaluronic acid gels according to strength, flexibility, and associated clinical significance. J Drugs Dermatol.

[16] Uppada UK, Tauro DP, Senthilnathan KP (2024). Cephalometric analysis of Indian races: a systematic review. Natl J Maxillofac Surg.

[17] Vashi NA, Buainain De Castro Maymone M, Kundu RV (2016). Aging differences in ethnic skin. J Clin Aesthet Dermatol.

[18] Shome D, Vadera S, Khare S, Ram MS, Ayyar A, Kapoor R, Desai N (2020). Aging and the Indian face: an analytical study of aging in the Asian Indian face. Plast Reconstr Surg Glob Open.

[19] Goodman GJ (2015). The oval female facial shape - a study in beauty. Dermatol Surg.

[20] Braz A, Eduardo CC (2020). The facial shapes in planning the treatment with injectable fillers. Indian J Plast Surg.

[21] Shetty R (2015). Outer circle versus inner circle: special considerations while rejuvenating an Indian face using fillers. J Cutan Aesthet Surg.

[22] Swift A, Liew S, Weinkle S, Garcia JK, Silberberg MB (2021). The facial aging process from the "inside out". Aesthet Surg J.

[23] Arsiwala SZ (2018). Simplifying injectables for volumetric rejuvenation of face. J Cutan Aesthet Surg.

[24] Uppada UK, Tauro DP, Senthilnathan KP (2024). Anthropometric analysis of Indian faces: a systematic review. J Maxillofac Oral Surg.

[25] Winiarska N, Stachura A, Roszkowski B, Pietruski P, Włodarski P, Paskal W (2024). Anthropometry and current aesthetic concept of the lower third of the face and lips in Caucasian adult population: a systematic review and meta-analysis. Aesthetic Plast Surg.

[26] Kollipara R, Walker B, Sturgeon A (2017). Lip measurements and preferences in Asians and Hispanics: a brief review. J Clin Aesthet Dermatol.

[27] Arian H, Alroudan D, Alkandari Q, Shuaib A (2023). Cosmetic surgery and the diversity of cultural and ethnic perceptions of facial, breast, and gluteal aesthetics in women: a comprehensive review. Clin Cosmet Investig Dermatol.

[28] Nouveau S, Agrawal D, Kohli M, Bernerd F, Misra N, Nayak CS (2016). Skin hyperpigmentation in Indian population: insights and best practice. Indian J Dermatol.

[29] Sarkar R, Ranjan R, Garg S, Garg VK, Sonthalia S, Bansal S (2016). Periorbital hyperpigmentation: a comprehensive review. J Clin Aesthet Dermatol.

[30] Ghatge AS, Ghatge SB (2023). The effectiveness of injectable hyaluronic acid in the improvement of the facial skin quality: a systematic review. Clin Cosmet Investig Dermatol.

[31] Milutinovic J, Zelic K, Nedeljkovic N (2014). Evaluation of facial beauty using anthropometric proportions. ScientificWorldJournal.

[32] Singh P, Birkett L, Dhar S, Krumhuber E, Mosahebi A, Ponniah A (2024). Facial beauty and the correlation of associated attributes: an empirical aesthetic database study. Plast Reconstr Surg Glob Open.

[33] Albert G, Wells E, Arnocky S, Liu CH, Hodges-Simeon CR (2021). Observers use facial masculinity to make physical dominance assessments following 100-ms exposure. Aggress Behav.

[34] Goldie K, Kerscher M, Fabi SG (2021). Skin quality - a holistic 360° view: consensus results. Clin Cosmet Investig Dermatol.

[35] Martschin C, Bahhady R, Li J (2025). Development and validation of a novel holistic Skin Quality Assessment Scale. J Cosmet Dermatol.

[36] Nikolis A, Avelar LE, Haddad A (2024). Turn your AART into a HIT using a complete range of aesthetic injectables: methodology for combining products to maximise patient outcomes. Clin Cosmet Investig Dermatol.

[37] Jain R, Huang P, Ferraz RM (2017). A new tool to improve delivery of patient-engaged care and satisfaction in facial treatments: the Aesthetic Global Ranking Scale. J Cosmet Dermatol.

[38] (2024). Hyaluronic acid vs collagen - what’s best?. https://www.galdermaaesthetics.com/which-is-better-hyaluronic-acid-or-collagen.

[39] Rogerio V, Germani Vieira M, Rabelo V, Carbone AC, Filho DA, da Silva AM, De la Torre Canales G (2022). Features to consider for mimicring tissues in orofacial aesthetics with optimal balance technology and non-animal stabilized hyaluronic acid (Restylane®): the MIMT concept. J Stomatol Oral Maxillofac Surg.

[40] Nikolis A, Enright KM, Cotofana S, Nguyen Q, Raco L, Weiner S (2024). Intracorporeal evaluation of hyaluronic acid fillers with varied rheological properties and correlations with aesthetic outcomes. Skin Res Technol.

[41] Yi KH, Winayanuwattikun W, Kim SY (2024). Skin boosters: definitions and varied classifications. Skin Res Technol.

[42] Yi KH, Park MS, Ree YS, Kim HM (2023). A review on “skin boosters”: hyaluronic acid, poly-L-lactic acid and pol-D-lactic acid, polydeoxyribonucleotide, polynucleotides, growth factor, and exosome. Korean Assoc Laser Dermatology Trichology.

[43] Hruza GJ (2007). Restylane stimulates collagen production. NEJM J Watch.

[44] Fagien S, Bertucci V, von Grote E, Mashburn JH (2019). Rheologic and physicochemical properties used to differentiate injectable hyaluronic acid filler products. Plast Reconstr Surg.

[45] De Boulle K, Glogau R, Kono T (2013). A review of the metabolism of 1,4-butanediol diglycidyl ether-crosslinked hyaluronic acid dermal fillers. Dermatol Surg.

[46] Shin SH, Lee YH, Rho NK, Park KY (2023). Skin aging from mechanisms to interventions: focusing on dermal aging. Front Physiol.

[47] Benar H, Benar EB (2024). A new nonsurgical combination approach for skin tightening and remodeling; Endoskin - a comparative study. J Cosmet Dermatol.

[48] Gubanova EI, Starovatova PA, Rodina MY (2015). 12-month effects of stabilized hyaluronic acid gel compared with saline for rejuvenation of aging hands. J Drugs Dermatol.

[49] Wu Y, Tian Y, Xu J, Zhong S, Wang R, Wu W (2020). A randomized study showing improved skin quality and aesthetic appearance of dorsal hands after hyaluronic acid gel treatment in a Chinese population. J Cosmet Dermatol.

[50] Voegeli R, Cherel M, Schoop R, Rawlings AV (2022). A comprehensive comparison of facial skin hydration based on capacitance and conductance measurements in Chinese women. Int J Cosmet Sci.

[51] Nikolis A, Enright KM (2018). Evaluating the role of small particle hyaluronic acid fillers using micro-droplet technique in the face, neck and hands: a retrospective chart review. Clin Cosmet Investig Dermatol.

